# Supplementary material for: Distinct functional constraints driving conservation of the cofilin N-terminal regulatory tail
Source: Nat Commun. 2024 Feb 16;15:1426. doi: 10.1038/s41467-024-45878-9 (PMC10873347; doi:10.1038/s41467-024-45878-9)

Supplementary Information

Distinct functional constraints driving conservation of the cofilin N-terminal regulatory tail

Joel A. Sexton[1], Tony Potchernikov[2], Jeffrey P. Bibeau[2], Gabriela Casanova-Sepúlveda[2], Wenxiang Cao[2], Hua Jane Lou[1], Titus J. Boggon[1,2], Enrique M. De La Cruz[2], and Benjamin E. Turk[1,*]

[1]Department of Pharmacology, Yale School of Medicine, New Haven, CT 06520
[2]Department of Molecular Biophysics and Biochemistry, Yale University, New Haven, CT 06520

*Corresponding author. Email: ben.turk@yale.edu

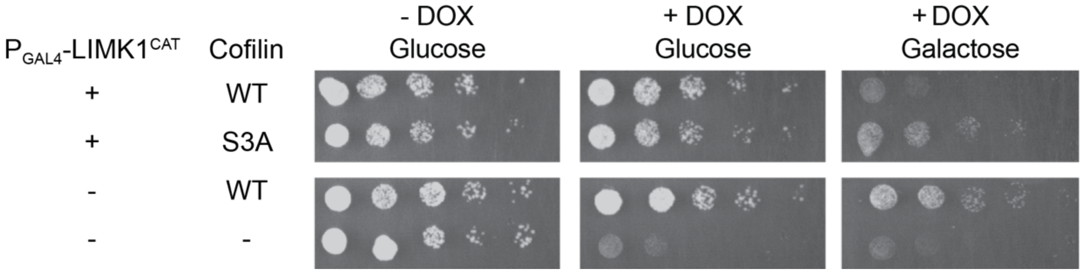

Supplementary Fig. 1. Growth inhibition of yeast by galactose-induced LIMK1^{CAT} expression. Yeast harboring the indicated expression plasmids were grown on either glucose or galactose to induce LIMK1^{CAT} expression in the presence or absence of doxycycline (DOX). Image representative of *n*=7 independent experiments.

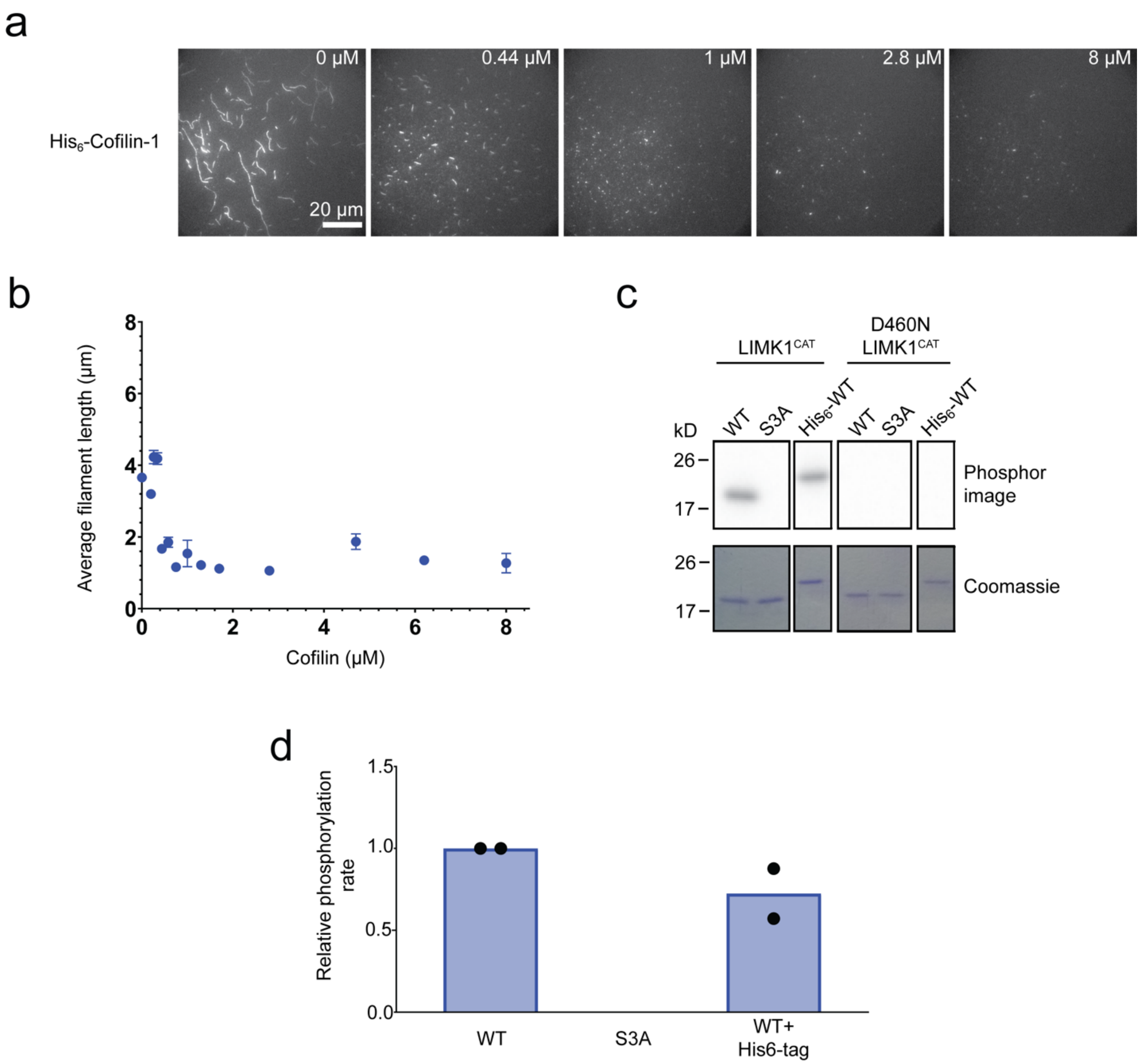

Supplementary Fig. 2. N-terminal His$_6$-tagged cofilin can sever actin and be phosphorylated by LIMK1. a TIRF microscopy images of Alexa647-labeled actin filaments incubated with N-terminally His$_6$-tagged cofilin-1. Cofilin concentrations listed in upper right corner of each image. The experiment was performed once. **b** Quantification of actin filament length at the indicated concentrations of His$_6$-tagged cofilin-1. Data points show average length, and error bars show SD for all filaments imaged in a single experiment. The numbers of filaments (*n*) quantified for each concentration are provided in the Source Data file and ranged from 140 – 1343. **c** In vitro radiolabel kinase assay comparing phosphorylation of purified 2 µM tagged and un-tagged cofilin^{WT} and cofilin^{S3T} by 2 nM LIMK1 kinase domain for 10 min at 30 °C. Kinase inactive mutant (D460N) was assayed alongside. A representative two independent experiments is shown. **d** Quantification of phosphorylation rates normalized to untagged cofilin^{WT}. Bars show the mean of two independent experiments. Source data are provided as a Source Data file.

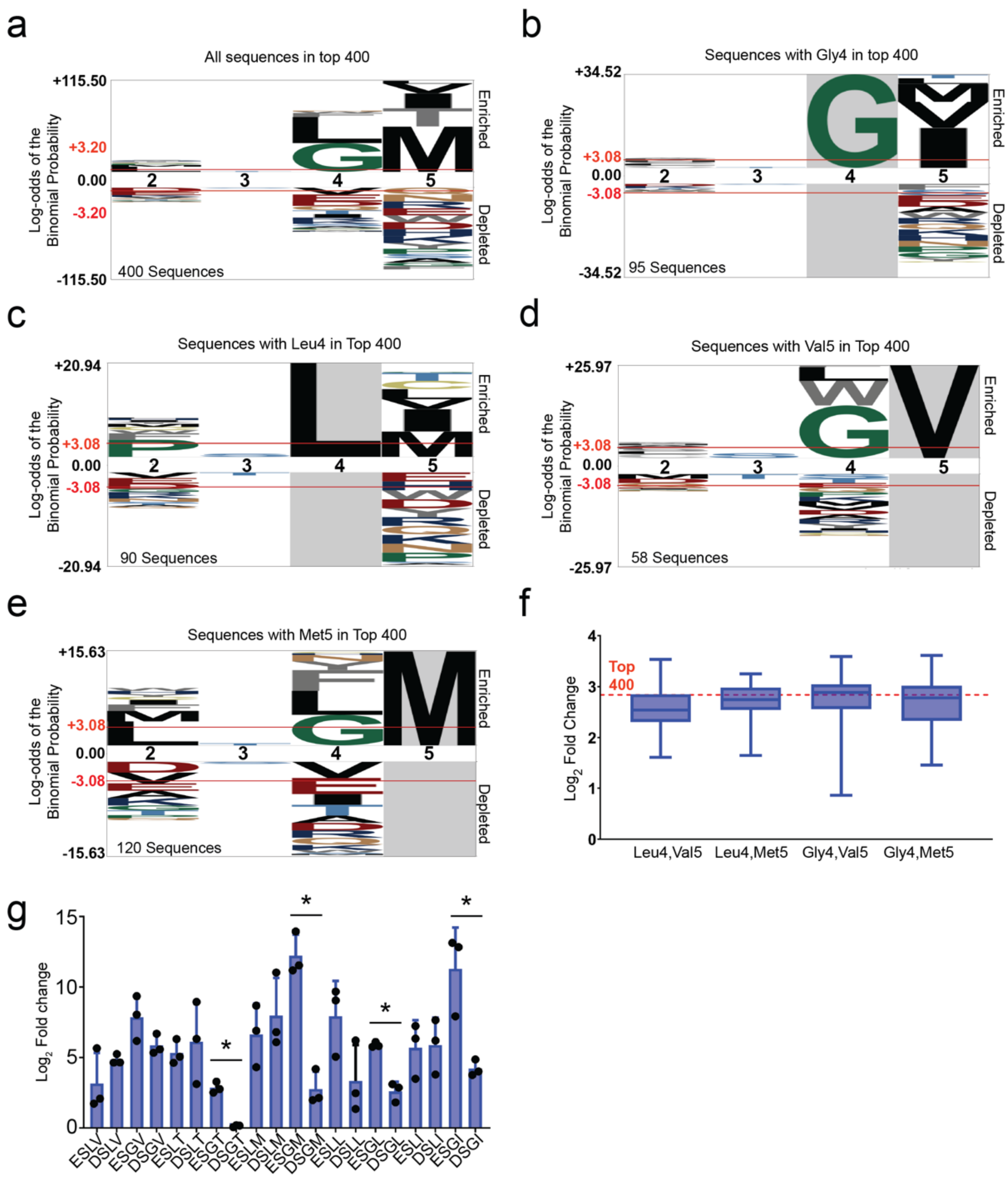

Supplementary Fig. 3. Cofilin N-terminus motifs identified in combinatorial library screen. a Sequence logo of the top 400 sequences based on average enrichment across 3 independently performed screens. Additional panels show logos of those sequences within the top 400 containing Gly4 (**b**), Leu4 (**c**), Val5 (**d**), or Met5 (**e**). The number of sequences (*n*) is at bottom left of each chart. Logos were generated using pLogo software[1], which calculates the significance threshold ($p = 0.05$, red lines) by a binomial probability function with Bonferroni correction. **f** Enrichment distribution of *n*=40 cofilin sequences containing Leu4-Val5, Leu4-Met5, Gly4-Val5, and Gly4-Met5 combinations of residues. Box plots indicate median (middle line), 25th and 75th percentile (box) and 10th and 90th percentile (whiskers). **g** Mean enrichment of selected sequences. Data are presented as mean values ± SD for the three independent screens. *, $p < 0.05$ by unpaired two-tailed Welch's t-test (ESGT vs. DSGT, p=0.0056; ESGM vs. DSGM, p=0.0013; ESGL vs. DSGL, p=0.01; ESGI vs. DSGI, p=0.048). Source data is in **Supplementary Data 2**.

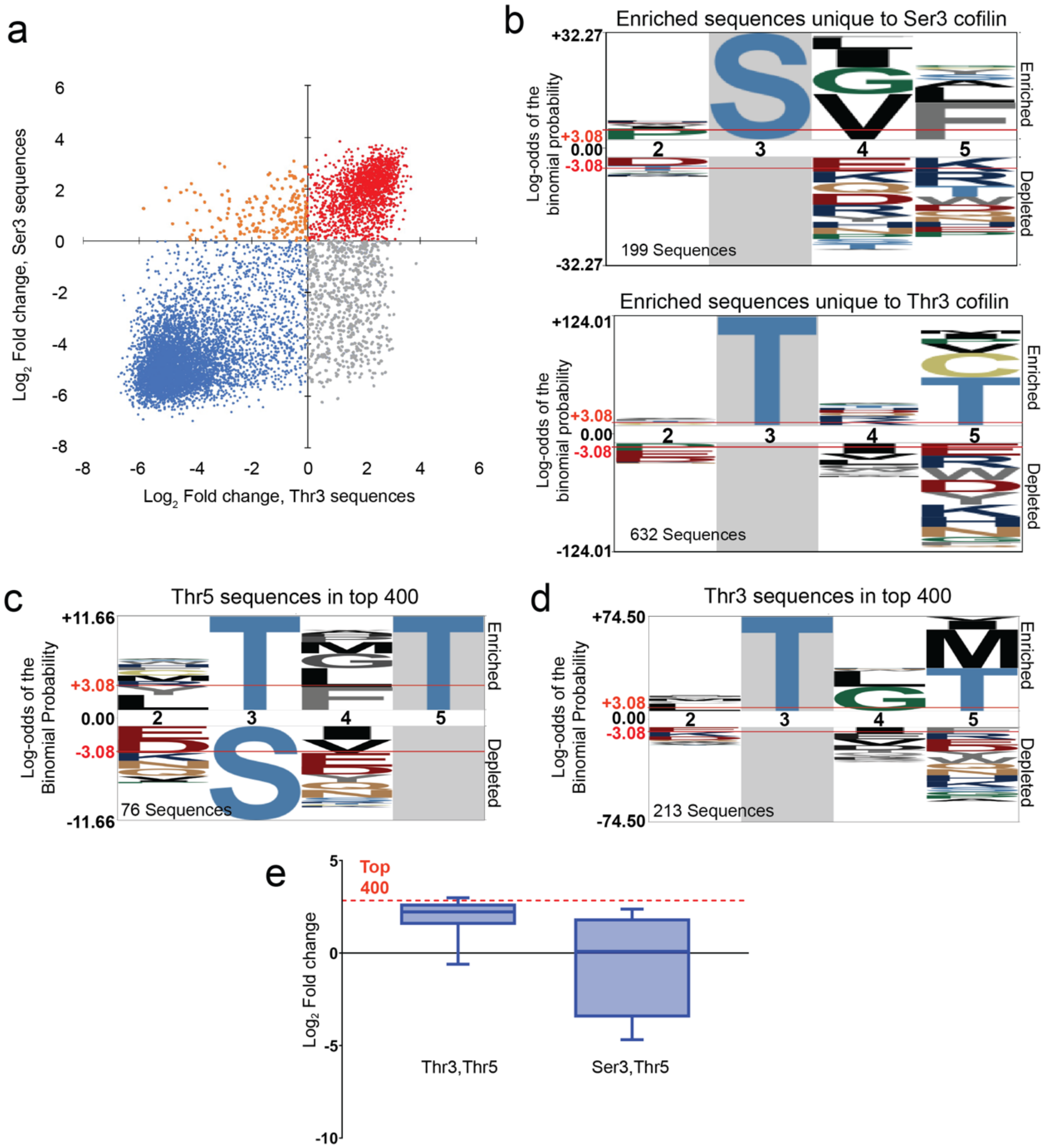

Supplementary Fig. 4. Functional cofilin sequences can depend on phospho-acceptor residue context. a Scatter plot showing enrichment of otherwise identical cofilin sequences with either a Ser3 or Thr3 residue. Average $\log_2$ fold change across the three replicate screens is shown for each sequence. **b** Sequence logo of uniquely enriched Ser3 (top, *n*=199) or Thr3 (bottom, *n*=632) cofilin variants. **c** Probability logo of the 400 most enriched sequences containing a Thr5 residue (*n*=76). **d** Probability logo of the 400 most enriched sequences containing a Thr3 residue (*n*=213). **e** Enrichment score distribution of cofilin sequences containing an x-T-x-T motif or an x-S-x-T motif. Each distribution includes *n*=400 sequences, and values are the average from three independent screens. Box plots indicate median (middle line), 25th and 75th percentile (box) and 10th and 90th percentile (whiskers). The red line shows the value for the 400th most enriched sequence from the entire library. For all pLogos, the significance threshold (*p* = 0.05, red lines) was calculated by a binomial probability function with Bonferroni correction. Source data is found in **Supplementary Data 2**.

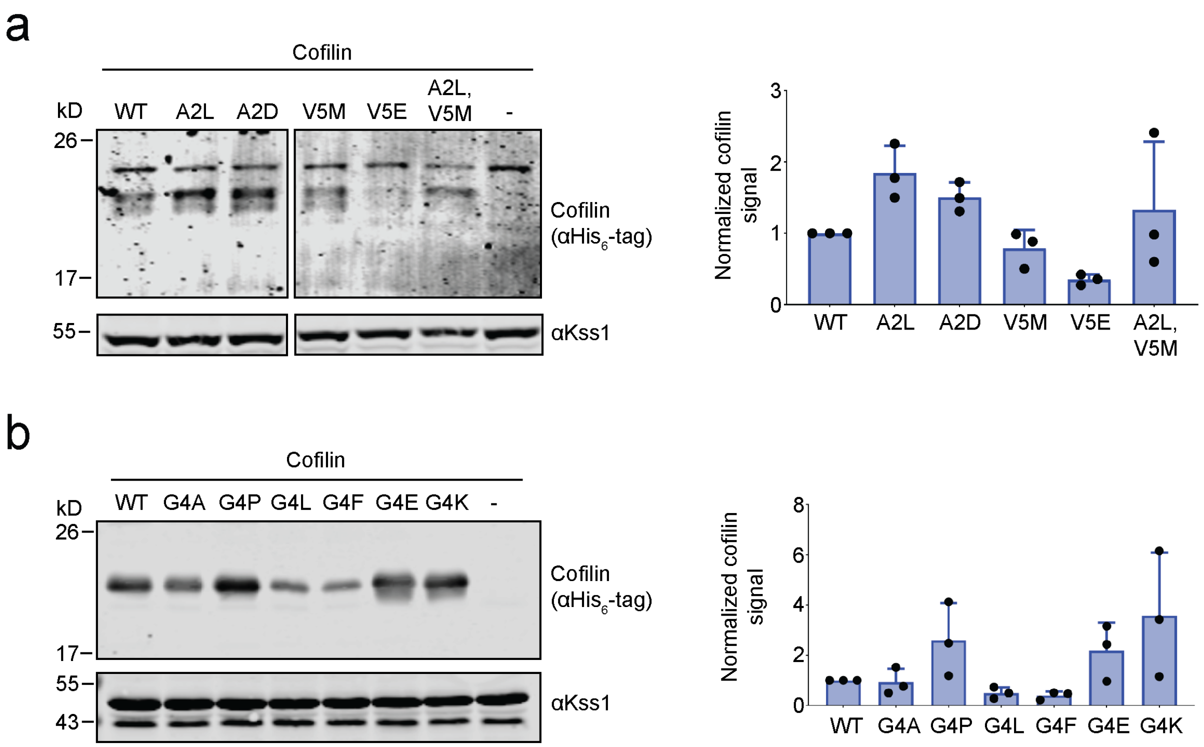

Supplementary Fig. 5. Protein expression levels of cofilin variants in yeast. a,b Immunoblots (left) and quantified protein levels (right) of cofilin protein levels in lysates corresponding to yeast plated in **Fig. 3a** (**a**) and **Fig. 3b** (**b**). Kss1 serves as a loading control. Quantified protein levels were normalized to the Kss1 signal as a fraction of the cofilin^{WT} signal. Data are presented as mean values ± SD for n=3 independent experiments. Source data are provided as a Source Data file.

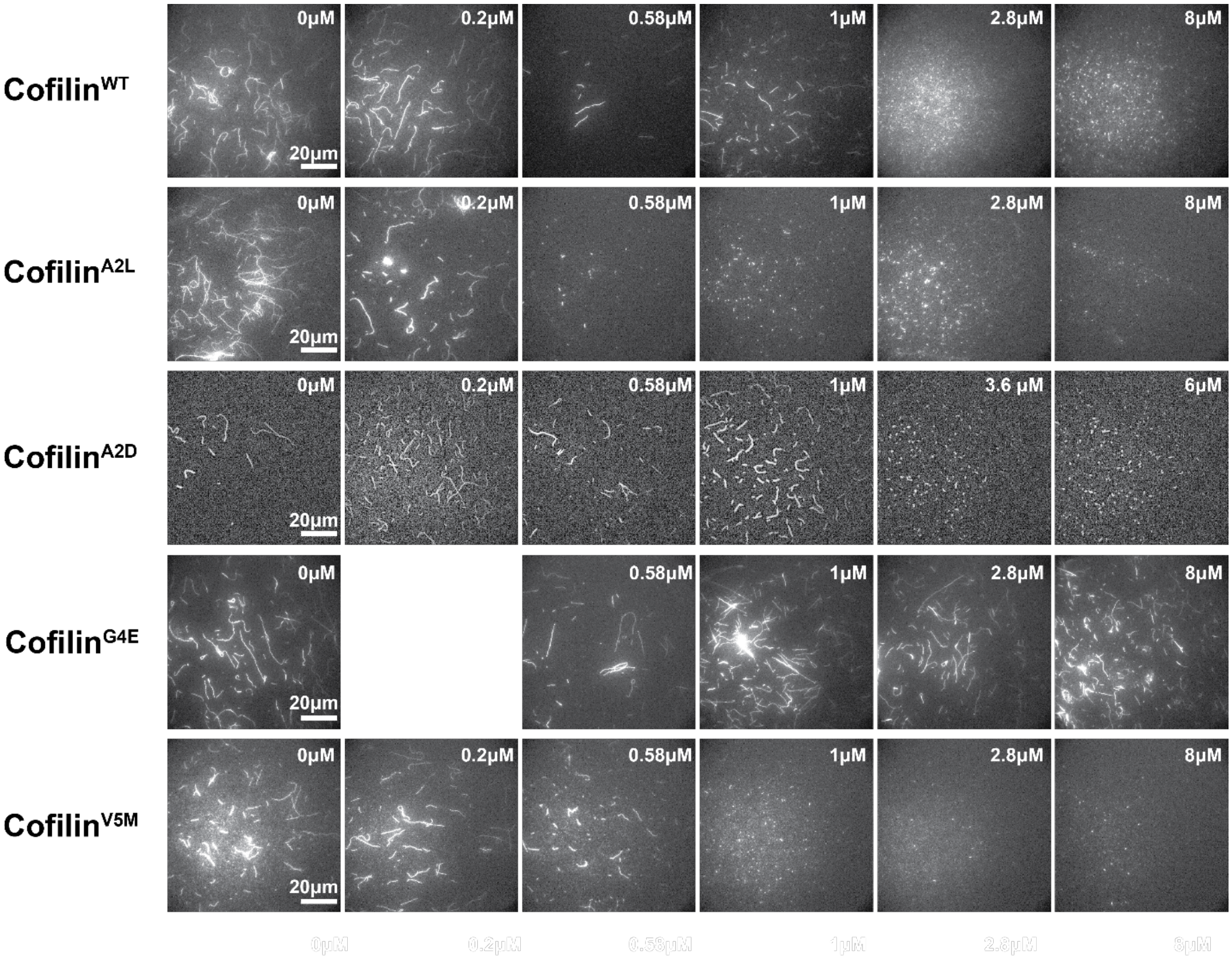

Supplementary Fig. 6. In vitro actin severing assays comparing cofilin mutants. TIRF microscopy images of Alexa647-labeled actin filaments incubated with the indicated concentration of WT or mutant cofilin. Representative images of 20 – 40 fields collected for each concentration are shown from a single replicate. This experiment was performed once.

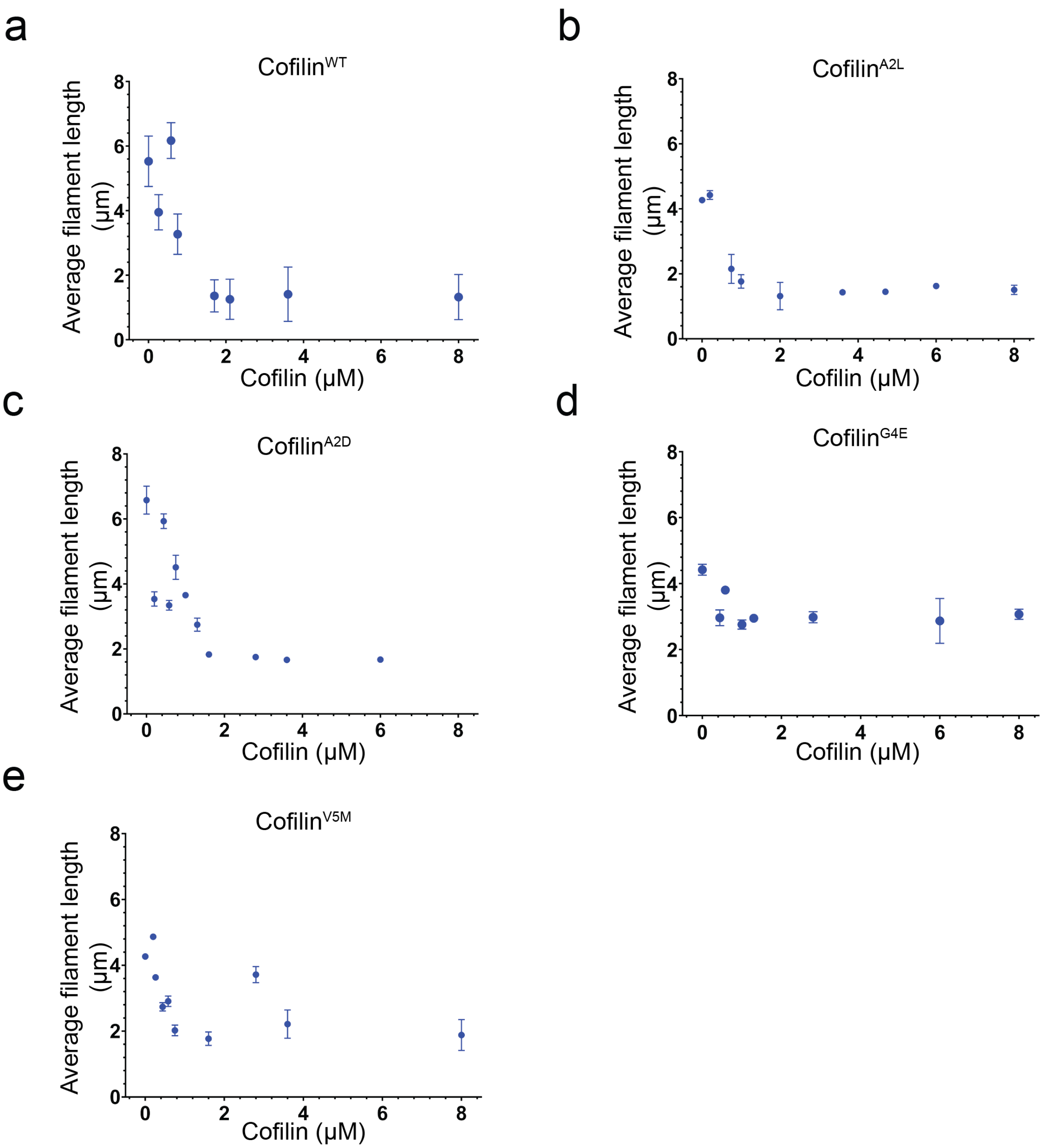

Supplementary Fig. 7. Quantification of in vitro actin severing assays comparing cofilin mutants. a-e Average Alexa647-labeled actin filament length following incubation with the indicated cofilin variants at various concentrations quantified from TIRF microscopy images, representatives of which are shown in **Supplementary Fig. 6**. Data points show average length, and error bars show SD for all filaments imaged in the single experiment performed. The number of filaments (*n*) quantified for each concentration ranged from 18 – 1897 and is given in the Source Data file.

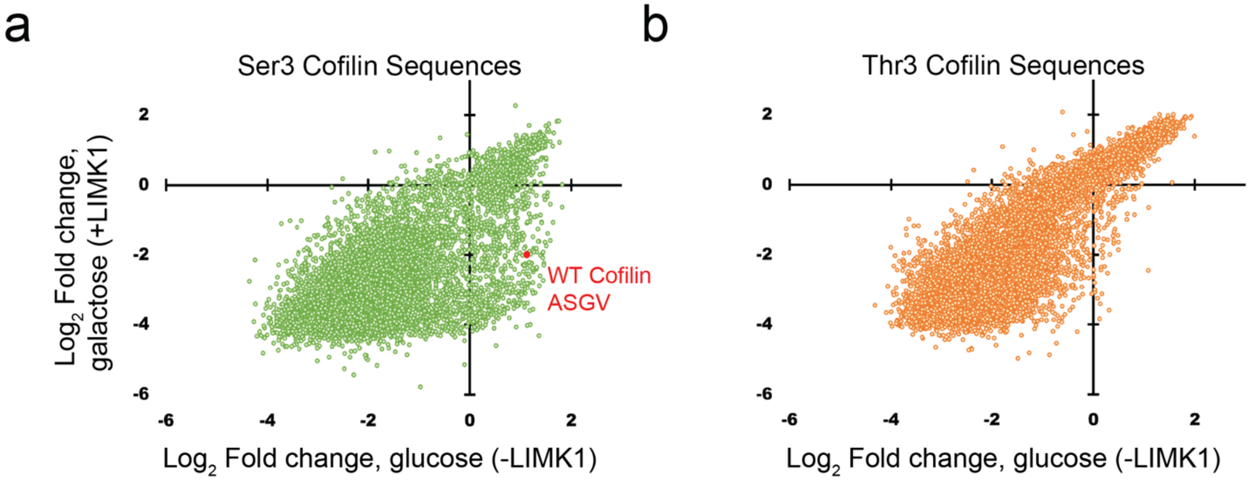

Supplementary Fig. 8. Distributions of Ser3 and Thr3 cofilin library sequences. a,b Plots show the change in relative abundance of Ser3 (**a**) and Thr3 (**b**) cofilin library sequences with and without LIMK1 induction. Each data point represents the average $\log_2$ fold change across three independently performed replicates. Source data are from **Supplementary Data 2**.

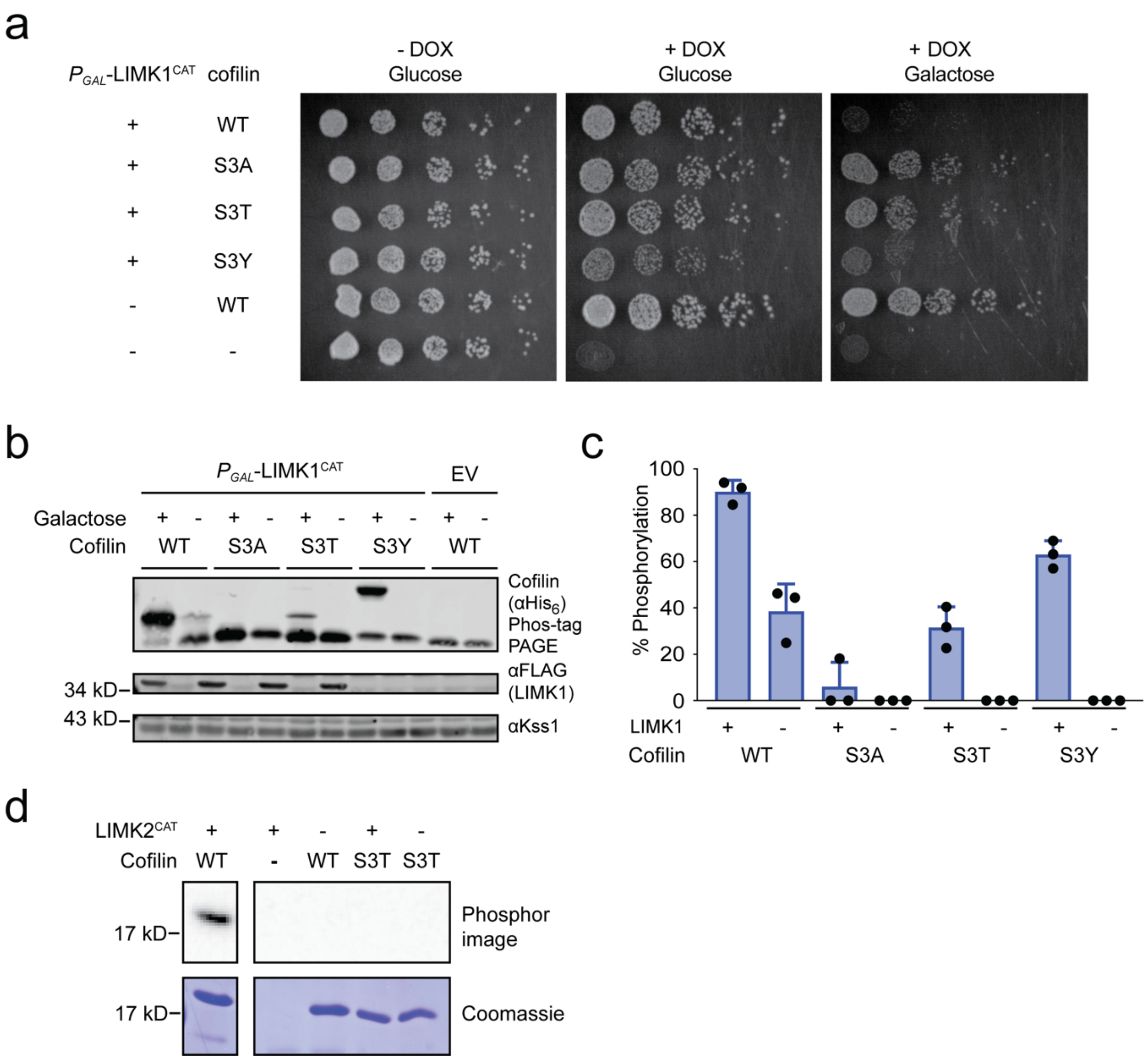

Supplementary Fig. 9. Effects of cofilin Ser3 substitutions on phosphorylation or regulation by LIMK catalytic domain constructs. a Growth of yeast expressing the indicated cofilin variants with (galactose) or without (glucose) induction of $LIMK1^{CAT}$. Representative of *n*=3 independent experiments. **b** Immunoblots of lysates corresponding to yeast plated in (**a**). Cofilin species are separated based on phosphorylation state by Phos-tag SDS-PAGE. Kss1 serves as a loading control. **c** Quantification of immunoblots in (**b**) showing the fraction of total cofilin phosphorylated. Data shown are mean ± SD for *n*=3 independent experiments. **d** Phosphorylation of WT or S3T cofilin by LIMK2 catalytic domain. Shown is a representative of *n*=2 independent experiments. Source data are provided as a Source Data file.

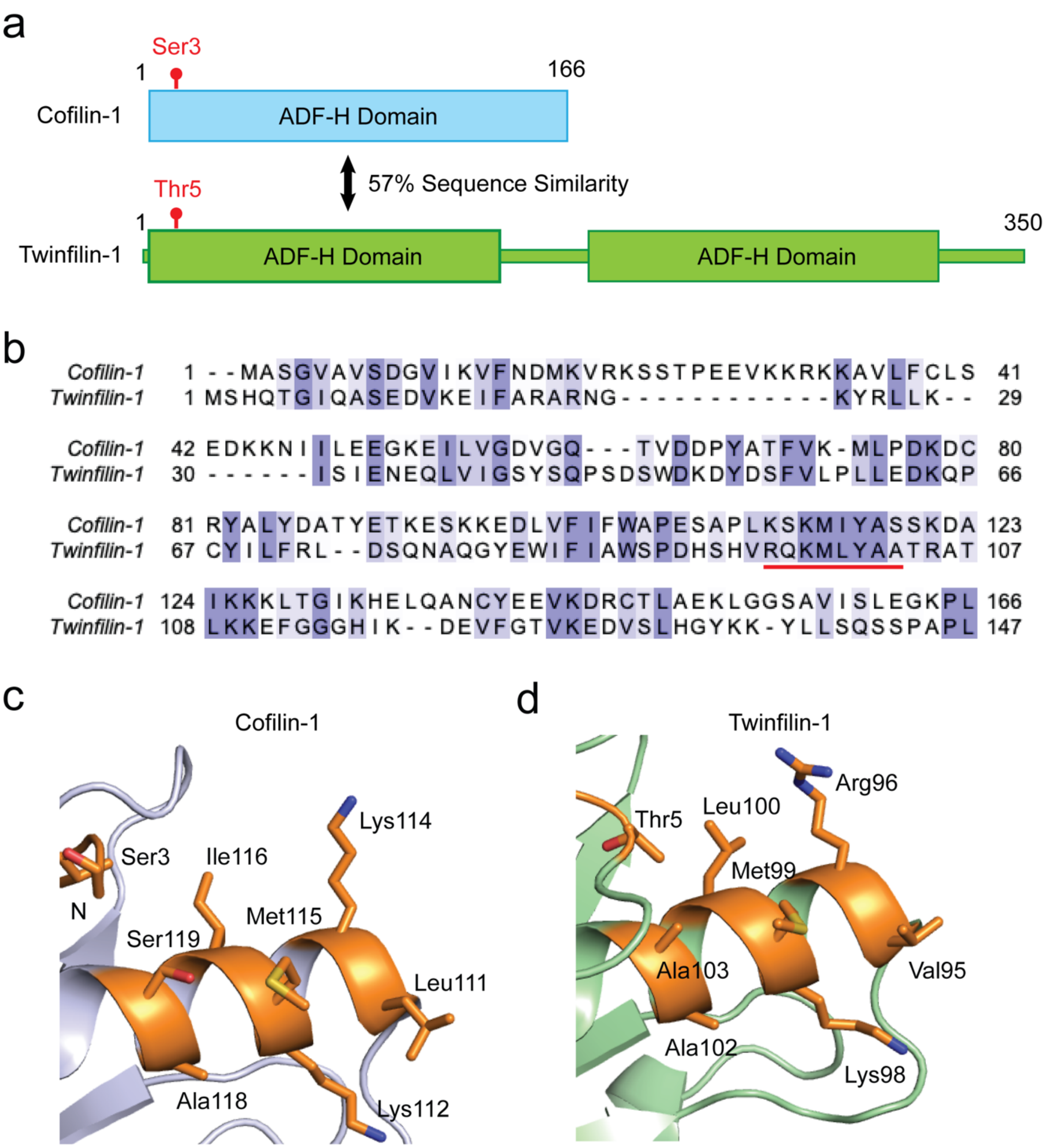

Supplementary Fig. 10. The LIMK binding interface of cofilin is conserved to twinfilin. a Diagram showing domain organization of human cofilin-1 and twinfilin-1. **b** Sequence alignment of human cofilin-1 and the N-terminal ADF-H domain of human twinfilin-1. The sequence at the LIMK-cofilin interface is underlined in red. **c** Cofilin residues (orange) (PDB ID: 5YU8)[2] at the LIMK1 binding interface. **d** Residues in the twinfilin-1 N-terminal ADF homology domain analogous to those at the LIMK1-cofilin interface (PDB ID: 7CCC)[3].

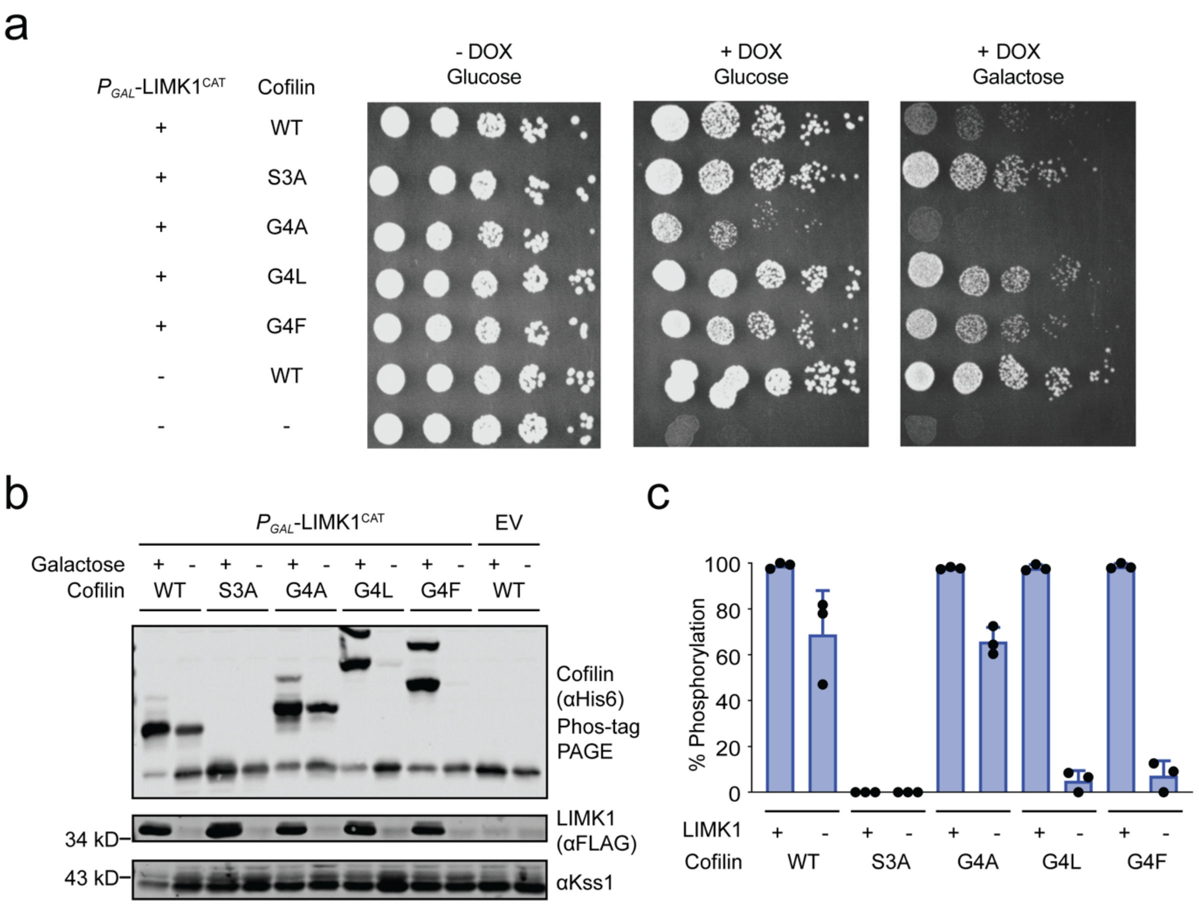

Supplementary Fig. 11. Exacerbated effects of LIMK1^{CAT} expression on growth inhibition in the context of cofilin Gly4 cofilin mutations. **a** Growth of yeast expressing the indicated cofilin variants with or without LIMK1^{CAT}. Representative of *n*=3 independent experiments. **b** Immunoblots of lysates corresponding to yeast plated in (**a**). Cofilin species are separated based on phosphorylation state by Phos-tag SDS-PAGE, with the slower migrating species corresponding to phosphorylated cofilin. **c** Fraction of cofilin phosphorylated in (**b**) from quantified immunoblots. Data show meant ± SD for *n*=3 independent experiments. Source data are provided as a Source Data file.

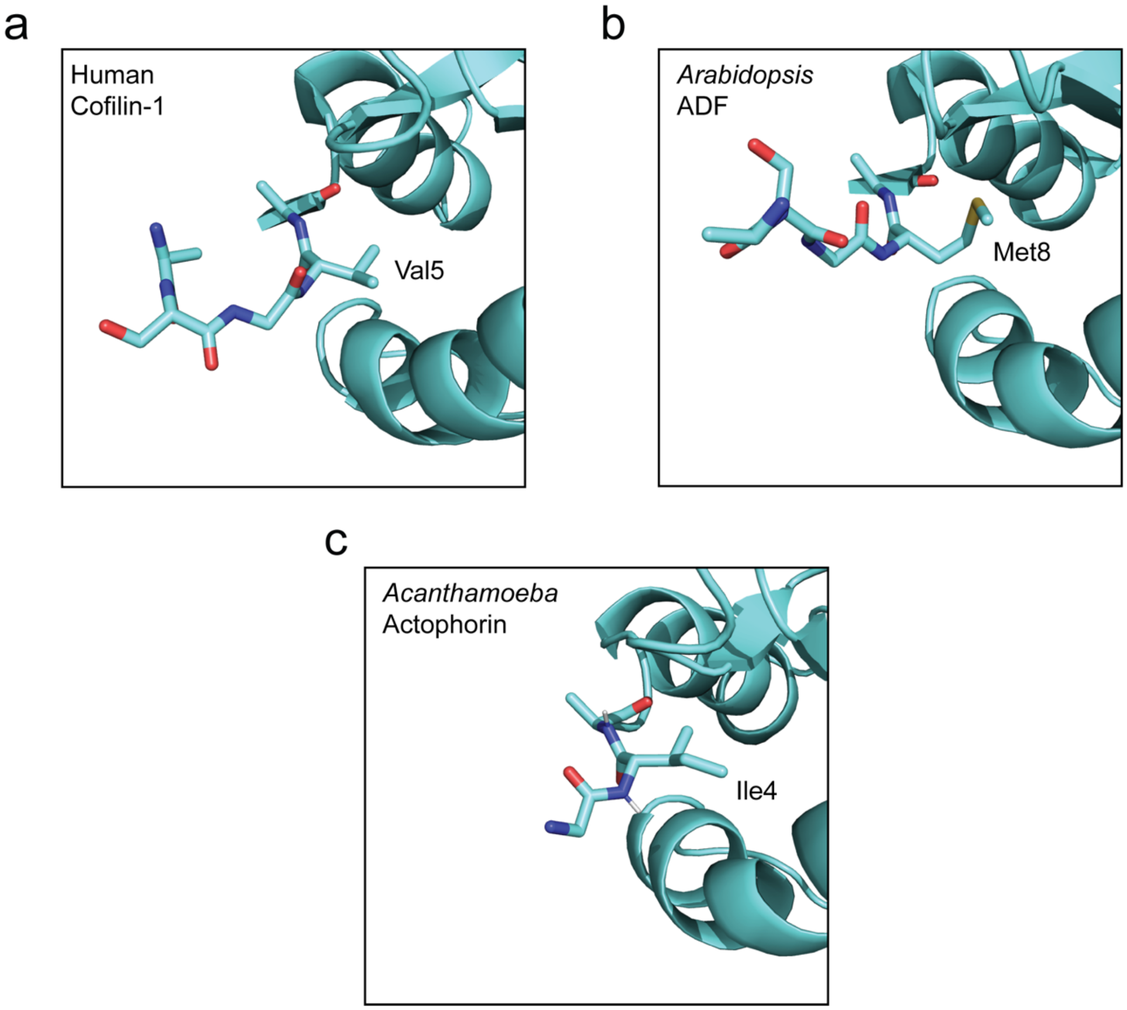

Supplementary Fig. 12. Comparison of N-terminal structures of cofilin orthologs. a-c X-ray crystal structures are shown for (**a**) human cofilin-1 (from its complex with LIMK1^{CAT}, PDB ID: 5HVK[4]), (**b**) *Arabidopsis* ADF (PDB ID: 1F7S[5]), and (**c**) *Acanthamoeba* actophorin (PDB ID: 1AHQ[6]).

a

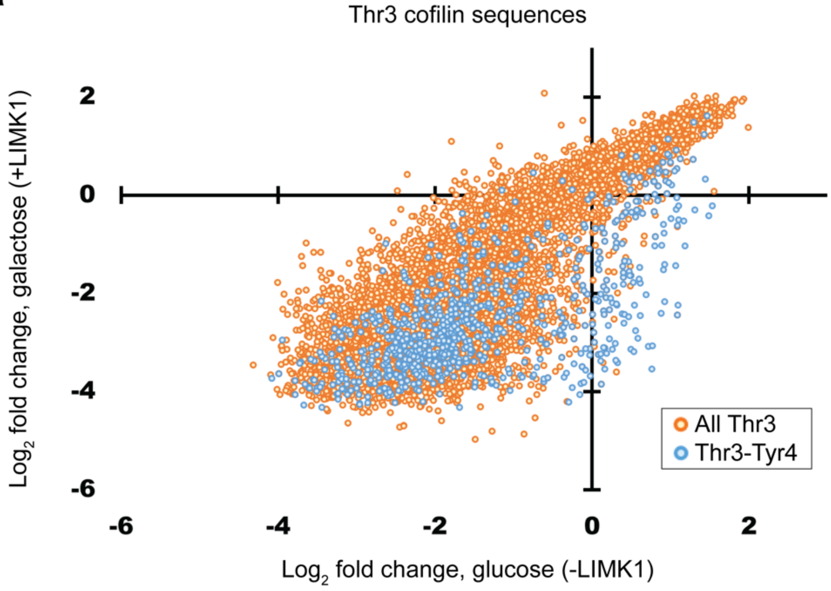

b

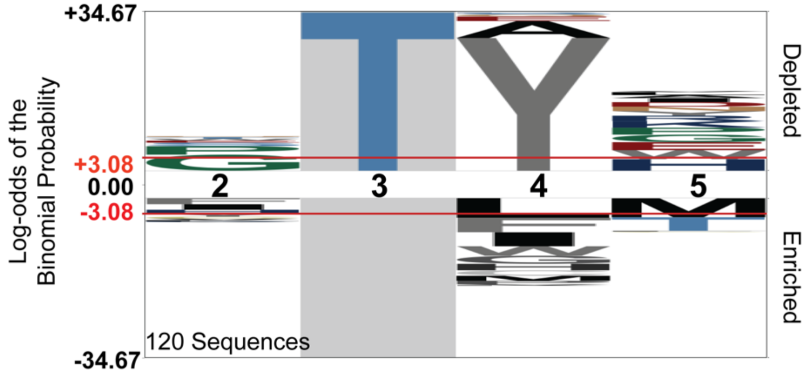

Supplementary Fig. 13. Distributions of x-T-Y-x cofilin library sequences. a Plot shows change in abundance of cofilin library sequences containing a x-T-Y-x motif (blue) compared to all sequences contain a Thr residue at position 3 (orange) grown with (galactose) or without (glucose) LIMK1 induction. Data show the average value across *n*=3 independently performed experiments. **b** Consensus sequence logo of *n*=120 cofilin sequences containing a Thr3 phosphoacceptor residue selectively depleted by LIMK1 induction (average log_2 fold change in galactose from three independently conducted screens <0) when compared to a background of all Thr3 cofilin sequences supporting yeast growth. The significance threshold (*p*=0.05, red lines) was calculated by a binomial probability function with Bonferroni correction. Source data is from **Supplementary Data 2**.

Supplementary references

1. O'Shea, J. P. *et al.* pLogo: a probabilistic approach to visualizing sequence motifs. *Nat. Methods* **10**, 1211-1212 (2013).

2. Tanaka, K. *et al.* Structural basis for cofilin binding and actin filament disassembly. *Nat. Commun.* **9**, 1860 (2018).

3. Mwangangi, D. M., Manser, E. & Robinson, R. C. The structure of the actin filament uncapping complex mediated by twinfilin. *Sci. Adv.* **7** (2021).

4. Hamill, S., Lou, H. J., Turk, B. E. & Boggon, T. J. Structural basis for noncanonical substrate recognition of cofilin/ADF proteins by LIM kinases. *Mol. Cell* **62**, 397-408 (2016).

5. Bowman, G. D. *et al.* A comparative structural analysis of the ADF/cofilin family. *Proteins* **41**, 374-384 (2000).

6. Leonard, S. A., Gittis, A. G., Petrella, E. C., Pollard, T. D. & Lattman, E. E. Crystal structure of the actin-binding protein actophorin from Acanthamoeba. *Nat. Struct. Biol.* **4**, 369-373 (1997).

Uncropped gel images from supplementary figures

Supplementary Fig. 2C:

Autorad

Coomassie

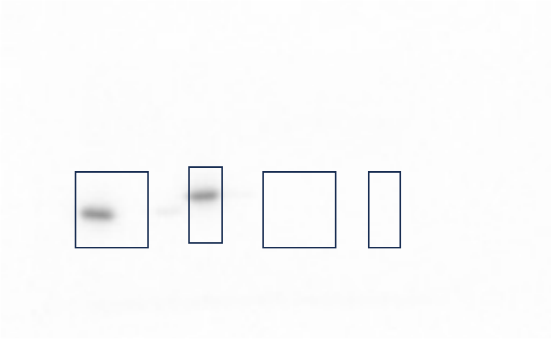

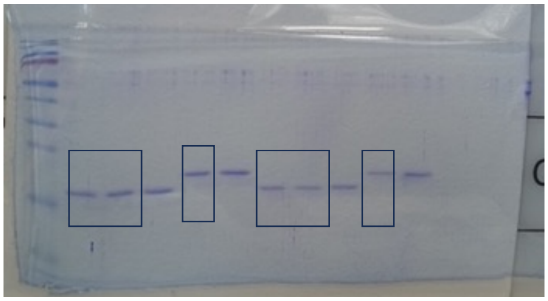

Supplementary Fig. 5A:

His$_6$

Kss1

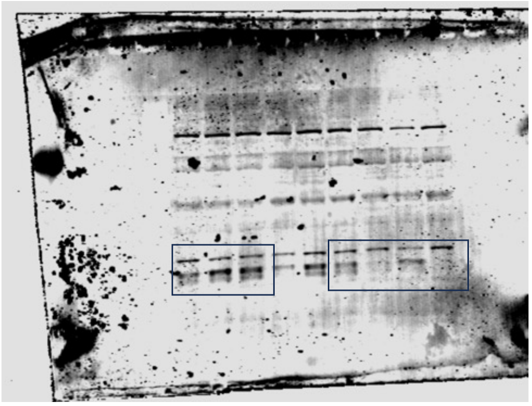

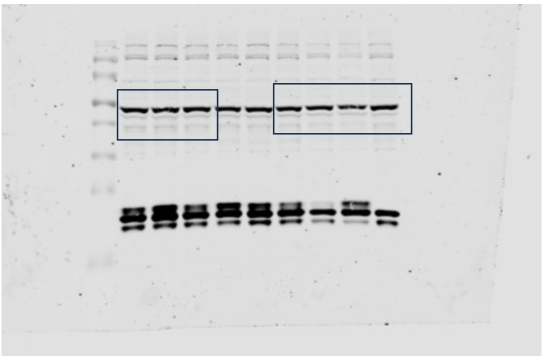

Supplementary Fig. 5B:

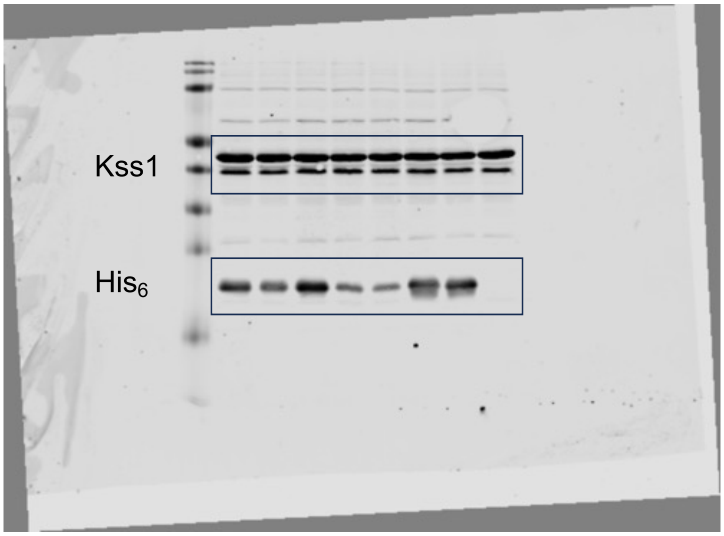

Supplementary Fig. 9B:

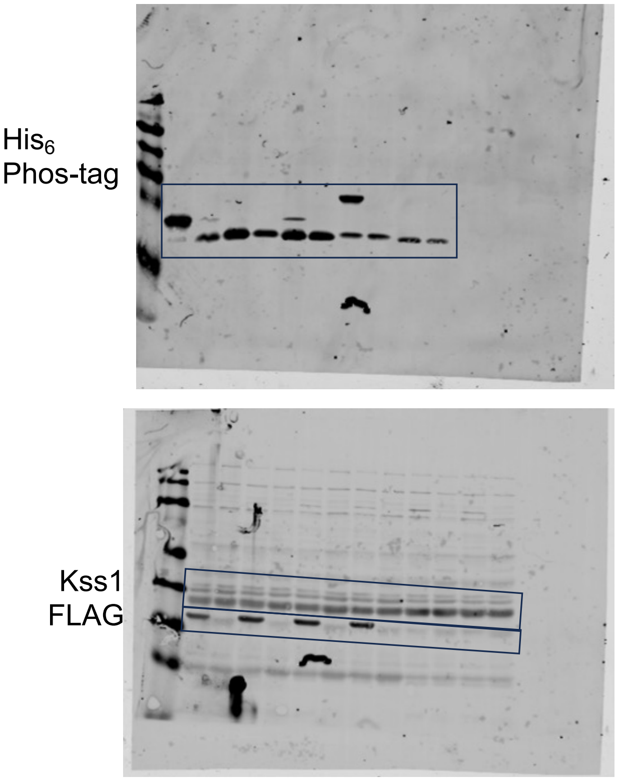

Supplementary Fig. 9D:

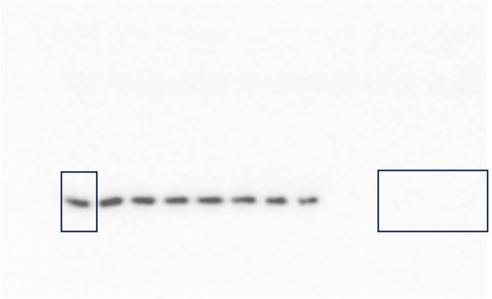

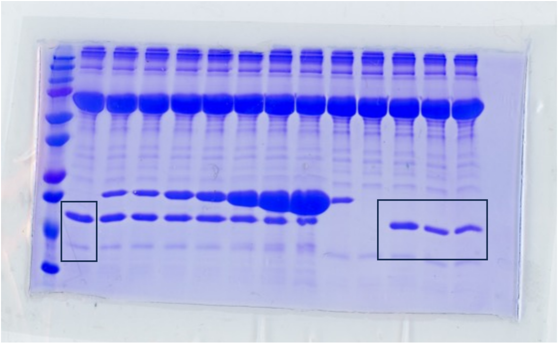

Supplementary Fig. 11B:

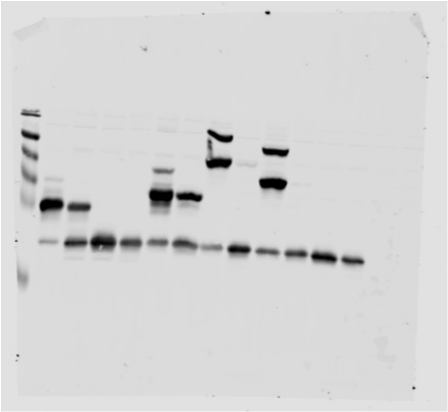

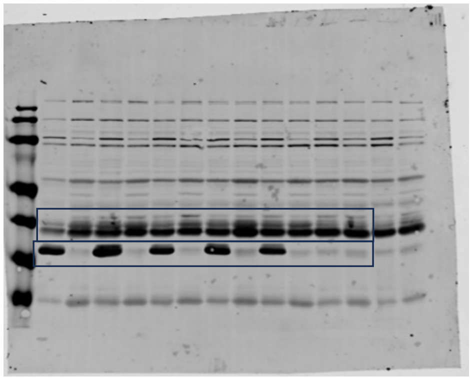

Supplement: Supplementary file 1 — Supplementary Information [file 41467_2024_45878_MOESM1_ESM.pdf]
