# Supplementary material for: Distinct functional constraints driving conservation of the cofilin N-terminal regulatory tail
Source: Nat Commun. 2024 Feb 16;15:1426. doi: 10.1038/s41467-024-45878-9 (PMC10873347; doi:10.1038/s41467-024-45878-9)
Supplement: Supplementary file 5 — Reporting Summary [file 41467_2024_45878_MOESM5_ESM.pdf]

nature portfolio

Corresponding author(s): Benjamin Turk

Last updated by author(s): 01-25-2024

Reporting Summary

Nature Portfolio wishes to improve the reproducibility of the work that we publish. This form provides structure for consistency and transparency in reporting. For further information on Nature Portfolio policies, see our Editorial Policies and the Editorial Policy Checklist.

Statistics

For all statistical analyses, confirm that the following items are present in the figure legend, table legend, main text, or Methods section.

n/a	Confirmed	
☐	☒	The exact sample size (*n*) for each experimental group/condition, given as a discrete number and unit of measurement
☐	☒	A statement on whether measurements were taken from distinct samples or whether the same sample was measured repeatedly
☐	☒	The statistical test(s) used AND whether they are one- or two-sided<br>*Only common tests should be described solely by name; describe more complex techniques in the Methods section.*
☒	☐	A description of all covariates tested
☒	☐	A description of any assumptions or corrections, such as tests of normality and adjustment for multiple comparisons
☐	☒	A full description of the statistical parameters including central tendency (e.g. means) or other basic estimates (e.g. regression coefficient) AND variation (e.g. standard deviation) or associated estimates of uncertainty (e.g. confidence intervals)
☐	☒	For null hypothesis testing, the test statistic (e.g. *F*, *t*, *r*) with confidence intervals, effect sizes, degrees of freedom and *P* value noted<br>*Give P values as exact values whenever suitable.*
☒	☐	For Bayesian analysis, information on the choice of priors and Markov chain Monte Carlo settings
☒	☐	For hierarchical and complex designs, identification of the appropriate level for tests and full reporting of outcomes
☒	☐	Estimates of effect sizes (e.g. Cohen's *d*, Pearson's *r*), indicating how they were calculated

Our web collection on statistics for biologists contains articles on many of the points above.

Software and code

Policy information about availability of computer code

Data collection	Image Studio 5.2.5 - immunoblot visualization
Data analysis	Image Studio 5.2.5 - immunoblot quantification<br>Microsoft Excel 16.75 - next generation sequencing data processing<br>pLogo - sequence logo generation<br>Graphpad Prism 9 - curve fitting<br>Image J 1.53a - TIRF microscopy image processing<br>Persistence version 4.2.3 - calculation of actin filament length<br>Image Lab - quantification of radiolabel kinase assays

For manuscripts utilizing custom algorithms or software that are central to the research but not yet described in published literature, software must be made available to editors and reviewers. We strongly encourage code deposition in a community repository (e.g. GitHub). See the Nature Portfolio guidelines for submitting code & software for further information.

Data

Policy information about availability of data

All manuscripts must include a data availability statement. This statement should provide the following information, where applicable:

- Accession codes, unique identifiers, or web links for publicly available datasets
- A description of any restrictions on data availability
- For clinical datasets or third party data, please ensure that the statement adheres to our policy

Next generation sequencing data is available through the Gene Expression Omnibus (GEO) repository with accession number GSE242403.

Research involving human participants, their data, or biological material

Policy information about studies with human participants or human data. See also policy information about sex, gender (identity/presentation), and sexual orientation and race, ethnicity and racism.

Reporting on sex and gender	N/A
Reporting on race, ethnicity, or other socially relevant groupings	N/A
Population characteristics	N/A
Recruitment	N/A
Ethics oversight	N/A

Note that full information on the approval of the study protocol must also be provided in the manuscript.

Field-specific reporting

Please select the one below that is the best fit for your research. If you are not sure, read the appropriate sections before making your selection.

☒ Life sciences ☐ Behavioural & social sciences ☐ Ecological, evolutionary & environmental sciences

For a reference copy of the document with all sections, see nature.com/documents/nr-reporting-summary-flat.pdf

Life sciences study design

All studies must disclose on these points even when the disclosure is negative.

Sample size	Sample sizes were chosen to ensure reproducibility. For most experiments, at least 3 independent replicates were performed as is standard for biochemistry and molecular biology experiments. For statistical analysis using unpaired t-tests, data were from experiments with n=3 independent experiments. Sample size for statistical analysis using pLogos used parameters inherent to the experiment: the library size of 16,000 sequences had all possible combinations of the 20 amino acids at three positions and two possible residues at one position (20^3 x 2 sequences), and the foreground sequence was dictated by the number of sequences enriched or depleted under a given set of conditions. Screens were conducted 3 times to ensure that statistical tests could be performed for pairwise comparison of variants. Experiments in which statistical tests were performed had n of at least 3. For actin binding assays, several samples that we interpret qualitatively had only two replicates. For actin severing assays the number of filaments analyzed was chosen in part to avoid bias (all filaments in a given field were counted), and enough fields were counted to have n>100 filaments if possible to ensure a normal distribution of filament size.
Data exclusions	No data were excluded from any analyses.
Replication	The number of independently conducted replicates performed for each experiments is indicated in the figure legends.
Randomization	Samples were not randomized since randomization is not appropriate for the experiments conducted here..
Blinding	Samples were not blinded as this is not appropriate for most of our experiments that had immunoblotting or autoradiographic gel readouts in which sample order is determined by the parameters of the experiment. Other experiments used automated analysis (plate reader or microscopic analysis). The yeast-based screens were effectively blinded as analysis was done by a core facility without knowledge of the nature of the samples.

Reporting for specific materials, systems and methods

We require information from authors about some types of materials, experimental systems and methods used in many studies. Here, indicate whether each material, system or method listed is relevant to your study. If you are not sure if a list item applies to your research, read the appropriate section before selecting a response.

Materials & experimental systems

n/a	Involved in the study
☐	☒ Antibodies
☒	☐ Eukaryotic cell lines
☒	☐ Palaeontology and archaeology
☒	☐ Animals and other organisms
☒	☐ Clinical data
☒	☐ Dual use research of concern
☒	☐ Plants

Methods

n/a	Involved in the study
☒	☐ ChIP-seq
☒	☐ Flow cytometry
☒	☐ MRI-based neuroimaging

Antibodies

Antibodies used

anti-penta-His (Qiagen #34650)
anti-FLAG M2 (Sigma #F3165)
anti-Kss1 (Santa Cruz #sc-6775-R)

Validation

anti-His and anti-FLAG antibodies are to epitope tags and do not react against endogenous proteins, and they were validated by analyzing samples expressing empty vector. The anti-Kss1 antibody that we use as a loading control has been validated by other groups using a Kss1 knockout strain (see Winters and Pryciak, PMID: 29321252).
