# Supplementary material for: Distinct functional constraints driving conservation of the cofilin N-terminal regulatory tail
Source: Nat Commun. 2024 Feb 16;15:1426. doi: 10.1038/s41467-024-45878-9 (PMC10873347; doi:10.1038/s41467-024-45878-9)
Supplement: Supplementary file 6 — Description of Additional Supplementary Files [file 41467_2024_45878_MOESM6_ESM.pdf]

Title: Supplementary Data 1.
Description: Raw read counts for all conditions and timepoints.

Title: Supplementary Data 2.
Description: Normalized read counts and enrichment scores for all screens.

Title: Supplementary Data 3.
Description: Oligonucleotides used in this study.
